# Mental Health Classification and Care for Individuals With Intellectual Disability and Severe Challenging Behaviour: A Three‐Year Follow‐Up on Quality of Life

**DOI:** 10.1111/jar.70243

**Published:** 2026-05-12

**Authors:** Ilse Migchelsen, Yvette M. Dijkxhoorn, Leo M. J. de Sonneville, Hanna Swaab

**Affiliations:** ^1^ Clinical Neurodevelopmental Sciences Leiden University Leiden the Netherlands; ^2^ Ipse de Bruggen Zoetermeer the Netherlands; ^3^ Leiden Institute for Brain and Cognition Leiden the Netherlands

**Keywords:** challenging behaviour, intellectual disability, mental health, quality of life, support

## Abstract

**Background:**

Individuals with intellectual disability and severe challenging behaviour (CB) often need intensive support. The present study examines whether mental health classification (MHC) makes interventions aimed at improving quality of life (QoL) more effective.

**Method:**

A three‐year follow up of 122 men and 62 women with intellectual disability and severe CB, aged 18–70 years, compared number and type of support goals and development of QoL (San Martin Scale) and CB (Developmental Behaviour Checklist for Adults) of individuals with (*N* = 124) and without (*N* = 60) MHC.

**Results:**

MHC was associated with setting more support goals. This had no differential effect on the development of QoL or CB. Improvement in QoL was associated with lower initial QoL, higher cognitive functioning and a lower number of support goals set.

**Conclusion:**

MHC was not related to better outcomes. Overall, setting fewer support goals and higher cognitive functioning are positively related to the effectiveness of treatment.

## Lay Summary

- People with intellectual disabilities *and* severe challenging behaviour often require significant support to cope with daily life challenges. Both intellectual disability and challenging behaviour can compromise their quality of life. Little research is available on what information could be used to enhance the effectiveness of support for this group.
- The present study examines whether mental health classification adds to the effectivity of interventions aimed at improving quality of life in individuals with intellectual disability and severe challenging behaviour. 184 individuals with intellectual disability and severe challenging behaviour were followed over 3 years of treatment. Of 124 individuals, a mental health classification was known.
- It was found that the use of mental health classification did not lead to more favourable treatment outcomes over time.
- It was found that the use of mental health classification resulted in setting more support goals, but this did not contribute to the effectiveness of treatment. Overall, the results suggest that it is more effective to spend available time and means resources on fewer goals anyway, that is, by prioritising certain goals over others. Finally, results suggest that higher cognitive functioning is related to higher receptivity to treatment.

## 1 Introduction

Intellectual disability is characterized by limitations in intellectual functioning and problems in adaptive functioning (Schalock et al. 2021). The prevalence of challenging behaviour among people with intellectual disability varies between 10% and 19% (Bowring et al. 2019; Jones et al. 2008; Lowe et al. 2007; Lundqvist 2013) and an estimated 5%–10% show severe challenging behaviour (Emerson 2001). There might be a higher risk of challenging behaviour in cases of more severe or profound intellectual disability (Bowring, Totsika, Hastings, Toogood, and Griffith 2017; Koritsas and Iacono 2012). Challenging behaviour is defined as culturally abnormal behaviour of such intensity, frequency or duration that the physical safety of the individual or others is placed in serious jeopardy and results in limited use of or access to ordinary community facilities (Emerson 2001). Severe challenging behaviour is often highly persistent over time, reflected in treatment resistance and is associated with negative personal and social outcomes (Smith et al. 2022; Thompson et al. 2022; Totsika et al. 2008). Challenging behaviour in addition to (severe) intellectual disability often results in the need for intensive support for daily functioning and a (semi)residential professional living environment facilitating interventions to contribute to quality of life.

Quality of life is considered to be the result of a dynamic process that builds on the realisation of personal goals and on assets to optimise individual potential (Reinders and Schalock 2014; Schalock, Baker, et al. 2018). Challenging behaviour can compromise quality of life in individuals with intellectual disability by limiting social participation, reducing access to community activities, increasing experiences of exclusion and also the use of restrictive measures or psychotropic medication that might undermine personal wellbeing (Bowring, Totsika, Hastings, Toogood, and McMahon 2017; Emerson and Einfeld 2011; Jonker et al. 2024).

To improve quality of life, it is therefore crucial for decision making in care for persons with intellectual disability and challenging behaviour to understand the individual support needs in daily functioning (Lombardi et al. 2016). These needs are defined by type and intensity of support necessary to facilitate an individual's participation in activities that are associated with everyday human functioning (Thompson et al. 2009). Reducing the level of unmet needs, for example, by finding an appropriate living accommodation or optimising care for physical or mental health needs, is supposed to improve quality of life (Koch et al. 2015). Individual differences in type or intensity of challenging behaviour influences quality of life and therefore may result in different indications for support needs (Emerson 2001; Esteban et al. 2021; Gur 2018). Support needs and goals are usually described in individual support plans (ISPs) following multidisciplinary deliberation and aim to enhance (social) participation and consequently quality of life (Gur 2018; Lombardi et al. 2016; Schalock, van Loon, and Mostert 2018; Schalock et al. 2016; van Loon et al. 2013).

Effective support is considered essential to serve quality of life. This might be especially true when severe challenging behaviour accompanies the intellectual disability, acknowledging that individual needs often go beyond typical needs of intellectual disability alone (Schalock et al. 2021; Verhaar et al. 2024). Understanding challenging behaviour in individuals with intellectual disability might be essential in decision making about effective residential support.

Classification of challenging behaviour with the Diagnostic Statistic Manual (DSM) is a widely used method to explain behavioural characteristics and subsequently choose evidence‐based interventions that are known to be supportive for daily functioning and well‐being. Classification provides a framework for identification of clinical needs and supports evidence‐based clinical decision‐making concerning effective treatment. In care for individuals with intellectual disability and severe challenging behaviour, mental health problems are not always assessed according to a classification system. One could argue that classification systems are only partially suitable for people with intellectual disability, as they might overlook developmental nuances and risk inaccurate diagnoses (Deb et al. 2022). However, since mental health classifications are helpful in finding directions for effective interventions, it is important to evaluate whether information from mental health classification is useful in guiding the search for individual support needs and treatment goals to promote personal outcomes in individuals with intellectual disability and severe challenging behaviour.

The use of mental health classification (MHC) is therefore expected to reduce challenging behaviour and improve quality of life. Regarding the expectations about type and quantity of support goals, expectations are less certain. MHC can lead to specifying more support goals, for example, by providing additional points of focus. Conversely, MHC may also lead to a reduction of support goals when some support goals are considered less relevant or are deprioritized relative to others.

## 2 Method

### 2.1 Design

In the Netherlands, individuals with intellectual disability *and* severe challenging behaviour who require around‐the‐clock intensive support often live in specialised institutions that offer 24/7 daily support. This study is prompted by the desire to improve the support provided by specialised service organisations. Residents of the four participating service organisations who meet the criteria of intellectual disability and severe challenging behaviour at the level of scale 6 and 7 of the WLz care profiles in the Netherlands (Wet langdurige zorg 2015) were eligible for inclusion.

Data were collected in a large longitudinal study called ‘Systematic Client Support Result Evaluation’ (SCORE) in the Netherlands, examining the effectiveness of interventions for individuals with intellectual disability who receive intensive support due to severe challenging behaviour. The provided intensive support was evaluated in three waves over a 3‐year period: at time of inclusion (starting 2017) (T1), 1 year later (T2) and 3 years after inclusion (T3). Informed consent to participate in the SCORE project was obtained from either participants themselves, if they had the mental capacity to take an informed decision or (in most cases) from their legal representatives.

Ethical approval for the study was granted by the ethics committee of the Faculty of Social and Behavioural Sciences, Clinical Neurodevelopmental Sciences, Leiden University, (ECPW‐2015/094) and by the ethical committee of the largest participating health care organisation (Ipse de Bruggen).

### 2.2 Participants

All participants were living in campus‐style residential or village communities, belonging to service organisations specialised in 24/7 care for people with intellectual disability with a typical client‐staff ratio in direct caretaking of 4/3:1. Care managing clinical psychologists were asked to select those individuals who met the following criteria: of adult age with intellectual disability, living in staffed group homes and needing intensive support due to severe challenging behaviour. *N* = 543 potential participants were invited to join the study. *N* = 381 (70.2%) invitees responded, from whom *N* = 289 (75.9%) gave consent to participate. In the course of the study *N* = 57 participants dropped out for various reasons such as relocation, illness, death, planning problems with service staff team or withdrawal of consent by legal representative, resulting in a total of *N* = 232 participants. For the present study, another *N* = 48 participants were lost due to missing information about ISPs and/or lack of a recent intelligence test score, that is, less than 5 years prior to the time of data collection. The final sample consisted of *N* = 184 adults, aged 18 to 70 years (M = 40.23, SD = 14.21), including both males (*N* = 122; 66.3%) and females (*N* = 62; 33.7%).

### 2.3 Mental Health Classification

An individual support plan (ISP) contains all information caretakers need to support the participants on a daily basis and includes a current overview of all available diagnostic reports, a client description and other relevant information about individual needs and arrangements. The involved clinical psychologist is responsible for diagnosing mental health problems, using the DSM (American Psychiatric Association 2022) and the inclusion of this information in the ISP. It was verified whether a mental health classification (MHC), co‐occurring to the intellectual disability, was available in the ISP. A total of *N =* 124 (67.4%) individuals had one or more co‐occurring mental health classifications (MHC); age M = 39.97, SD = 14.3, 70.2% male and for *N =* 60 (32.6%) individuals there was no reference to any mental health classification (No MHC); M = 40.78, SD = 14.1, 58.3% male.

### 2.4 Level of Cognitive Functioning

Level of cognitive functioning was derived from ISPs and is based upon standardised developmental/IQ tests that were obtained within 5 years prior to data collection. In clinical practise, psychologists use different instruments, adapted to individual functioning (e.g., with respect to verbal and motor functioning.), such as: BSID‐II‐NL/Bayley‐III (van Baar et al. 2014), SON‐R, SON 2–8 (Tellegen and Laros 2011, 2017), WPPSI‐III‐NL, WISC‐III‐NL, WAIS III‐NL and WAIS IV‐NL (Wechsler 1989, 1991, 2008, 2012; Wechsler et al. 2011), with sufficient reliability and validity. All outcomes were converted to cognitive developmental age in months, referred to as level of cognitive functioning (CF).

### 2.5 Quality of Life

Quality of life was measured with the Dutch translation of the San Martin Scale (SMS), a multidimensional quality of life assessment scale for individuals with significant disabilities needing intensive and mostly complex support, suitable for all levels of intellectual disability (Verdugo et al. 2014). The SMS is a 95‐ items Likert scale questionnaire that provides a total score and eight domain scores, that is, self‐determination, emotional, physical and material well‐being, rights, personal outcomes, social inclusion and interpersonal relations. Questions are answered with never (1), sometimes (2), often (3) or always (4), with higher scores representing higher quality of life. Cronbach's alpha for the domains ranges from 0.82 to.93 and 0.97 for the total score (Verdugo et al. 2014). SMS total scores were converted to mean item scores (MIS).

### 2.6 Challenging Behaviour

Challenging behaviour was measured with the Developmental Behaviour Checklist for Adults (DBC‐A), a 107‐items questionnaire that assesses a comprehensive range of emotional, behavioural and mental health problems for adults with mild, moderate or severe levels of intellectual disability (Mohr et al. 2005). Next to a total score, six domains are distinguished, that is, Disruptive, Communication/anxiety, Antisocial, Self‐absorbed, Depressive and Social relating. Questions are answered with 0, 1 or 2 with higher scores representing higher frequencies of problem behaviour. Intraclass correlations for inter‐rater reliability and test–retest ranged from 0.72 to 0.85 and concurrent validity of emotional and behavioural disturbance is satisfactory (Mohr et al. 2005). DBC‐A total scores were converted to mean item scores (MIS).

At three moments in time, over a period of 3 years, that is, at the time of inclusion (T1), 1 year later (T2) and 3 years after inclusion (T3), SMS and DBC‐A were completed by a member of the participants core support team who was in the position to observe and interact frequently with the participant and knew the participant for at least 6 months. Due to planning problems, illness or decease, complete data sets of the three assessments were available for *N* = 137 (75%) individuals on the DBC‐A and *N* = 116 (63%) individuals on the SMS.

### 2.7 Support Goals

The individual's support and intervention requirements are formalised into an Individual Support Plan (ISP), drafted by the core support team. This team consists of members of the daily support staff and often includes the person with intellectual disability, their family members and/or legal representative, clinical psychologist and other involved practitioners. Goals serve as strategies for improvement of quality of life, can change over time and are evaluated at least yearly by the core support team.

For *N* = 184 participants, a total of 832 goals were identified. All goals, stated at T1, were assigned to one of the eight domains of the SMS, following a best matching procedure. By way of illustration, the following examples are given: Self‐determination goals intend to increase the autonomy of the individual and consider their opinions, personal preferences, decisions and choices. For example: ‘X decides for himself what he wants to eat and is able to get his daily groceries for his meals’. Emotional well‐being goals intend to enhance basic feelings of safety of the individual, satisfaction with life, the concept of self, emotional communication and absence of stress or negative feelings or behavioural problems. For example: ‘X experiences a meaningful weekend program and knows what activities to expect’. Physical well‐being goals intend to strengthen physical healthcare with topics such as nutrition, exercise, hygiene, mobility, medical service and sexuality. For example: ‘X has a healthy sleep pattern’. Material well‐being goals intend to improve work and household conditions, income, pension and technical aid. For example: ‘X can manage his expenses and save for Y’. Rights goals intend to increase individuals' knowledge of rights, intimacy, privacy, confidentiality and respect. For example: ‘X learns and understands his rights’. Personal development goals intend to enhance self‐improvement, learning, skills and motivational abilities. For example: ‘Within the next three months X learns how to prepare her own sandwich’. Social inclusion goals intend to improve integration, support and participation. For example: ‘X contributes to the residential area by doing odd small jobs in the area’. Interpersonal relations goals intend to strengthen social‐ and family relationships and communication. For example: ‘X receives a new communication system’. Goals that did not match with any of the eight domains, due to either mal formulation or because it was unclear what outcome it would represent, for example: ‘X adheres to his daily program’ or any reference to reducing or adding medication, restrictions of freedom or development of the support team, were excluded. A priori analysis, comparing the number of goals in this category between groups with and without mental health classification, demonstrated no difference (*p* = 0.124), indicating that this exclusion will not bias the results. A clinical psychologist matched all 832 goals with a quality of life domain, based upon described best matching procedure. To assess consistency in matching with the quality of life subdivisions, a sample of 100 goals was randomly selected and independently matched with a quality of life domain by two other behavioural specialists using the same best matching procedure. The inter‐rater reliability for three independent raters for this sample was calculated with Fleiss Kappa *ƙ* = 0.68, indicating a substantial agreement (Landis and Koch 1977).

The number of goals in the ISP ranged from 0 to 26 goals per individual (M = 4.52, SD = 4.84). A total of 665 goals were set for the individuals with one or more co‐occurring mental health classifications (MHC) and 167 goals were set for the individuals without mental health classifications (no‐MHC).

### 2.8 Statistical Analyses

All analyses were conducted in IBM SPSS Statistics version 27. Results were considered significant when *p* < 0.05.

#### 2.8.1 Preliminary Analyses

Due to planning problems, for example unavailability of staff within the time frame of planned research, illness or decease, complete data sets of the three assessments were available for *N* = 137 (75%) individuals on the DBC‐A and *N* = 116 (63%) individuals on the SMS. A priori *T*‐tests for independent samples were performed to verify whether individuals with at least one missing value on the DBC‐A or the SMS differed from individuals with complete data on DBC‐A and SMS total scores, level of cognitive functioning and (calendar) age. Similarly, a Chi‐square test was performed on gender distribution of groups with and without missing values. Results were as follows: there were no significant differences between these groups on distributions of sex, cognitive functioning, age and SMS‐total score at T1 (0.281 < *p* < 0.689). The group with incomplete data on the DBC‐A had a significantly higher score (M = 0.62, SD = 0.29) than the group with complete data (M = 0.52, SD = 0.21) at T1, [*t*(1,178) = 2.488, *p* = 0.015, *d* = 0.428].

To test initial differences between groups (MHC vs. No MHC) with complete data on the SMS at T1 *and* T2, on descriptive variables, a Chi Square Test on sex distribution and a *T*‐test for independent samples on level of cognitive functioning, age, DBC‐A and SMS total score at T1 were performed. On age, gender distribution, SMS and DBC‐A scores at T1, groups did not significantly differ (0.16 < *p* < 0.92). Cognitive functioning was significantly lower [T(135) = −1.856, *p* = 0.033, *d* = −0.346] in the group with MHC (61.22 ± 38.38 months) compared to the group without MHC (48.57 ± 31.70 months).

#### 2.8.2 Main Analyses

To explore whether MHC resulted in differences between groups in total number of support goals and distribution of goals focused on quality of life (QoL) domains at T1, repeated measures analyses of variance (RM ANOVAs) were performed, using ‘Type of goal’ (corresponding with the eight domains of quality of life) as within subject (WS) factor and Groups (MHC vs. no MHC) as between subjects (BS) factor, with number of support goals as dependent variable. Only individuals with complete SMS datasets at T1 and T2 were included in the analyses.

Differences between groups in challenging behaviour, respectively quality of life *over time*, were analysed using RM ANOVAs, with Time (T1‐inclusion vs. T2‐1 year later vs. T3‐3 years after inclusion) as a WS factor and group (MHC vs. no MHC) as a BS factor, with the DBC‐A total score (MIS) and SMS total score (MIS) as dependent variables, respectively. For the WS factor Time, a simple contrast was used with T2 vs. T1 as the first and T3 vs. T1 as the second contrast to evaluate shorter‐term and longer‐term changes, respectively. It is further explored whether the number of support goals is associated with changes in quality of life over time (QoL T2−QoL T1). As cognitive functioning appeared to be significantly correlated with challenging behaviour (DBC‐A) and quality of life (SMS) at T1, with *ρ*_s_ = −0.236 (*p* = 0.003, DBC‐A) and *ρ*_s_ = 0.339 (*p* < 0.001, SMS) respectively, a hierarchical multiple regression analysis was performed, separately for the groups, entering quality of life at T1 in the first step, cognitive functioning in the second step and total number of goals in the third step.

Furthermore, it was explored whether individuals who actually showed positive changes in quality of life from T1 to T2 (DeltaSMS ≥ 0) differed in number of goals and whether there were specific goals that contributed to changes in quality of life from those exhibiting negative changes in quality of life over time (DeltaSMS < 0). To this end, a multivariate analysis of variance (MANOVA), with Type of change in quality of life (positive: DeltaSMS ≥ 0, negative: DeltaSMS < 0) and Type of support goals as fixed factors, was performed to understand if there were specific goals that contributed to changes in quality of life over time.

Finally, to analyse whether total number of goals differed as a function of type of change in quality of life over time (positive vs. negative) and mental health classification (MHC vs. No MHC), a two‐way ANOVA was performed with Group (MHC vs. No MHC) and Type of Change (positive vs. negative) as fixed factors and number of goals as the dependent variable.

## 3 Results

### 3.1 Support Goals

**FIGURE 1:**
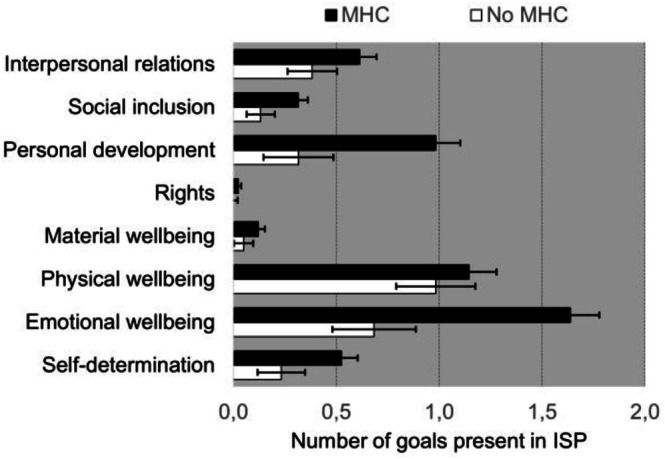
Mean number of individual goals (± SEM) in ISP as a function of group classification and type of targeted quality of life domain.

The RM ANOVA on number of individual goals per targeted QoL domain resulted in a significant main effect of Domain [*F*(7, 1274) = 34.505, *p* < 0.001, *η*_p_^2^ = 0.159], Group [*F*(1, 182) = 12.173, *p* < 0.001. *η*_p_^2^ = 0.063] and a Group × Domain interaction [*F*(7, 1274) = 5.101, *p* < 0.001. *η*_p_^2^ = 0.027]. These results (see Figure 1) indicate that (total) number of goals is larger in the group with a mental health classification, that the number of goals depend on type of domain and that differences between groups depend on type of Domain. Post hoc *T*‐tests for independent samples revealed that the number of goals in the MHC group compared to the No MHC group was significantly larger for the domains ‘self‐determination’ [*t*(178.204) = −2.505, *p* = 0.013, *d* = −327] ‘emotional well‐being’ [*t*(176.782) = −4.622, *p* < 0.001, *d* = −0.608], ‘personal development’ [*t*(179.657) = −4.132, *p* < 0.001, *d* = −0.507] and ‘social inclusion’ [*t*(177.298) = −2.591, *p = 0*.010, *d* = −0.34], while differences between groups on the other domains were not significant.

### 3.2 Changes in Quality of Life Over Time

**FIGURE 2:**
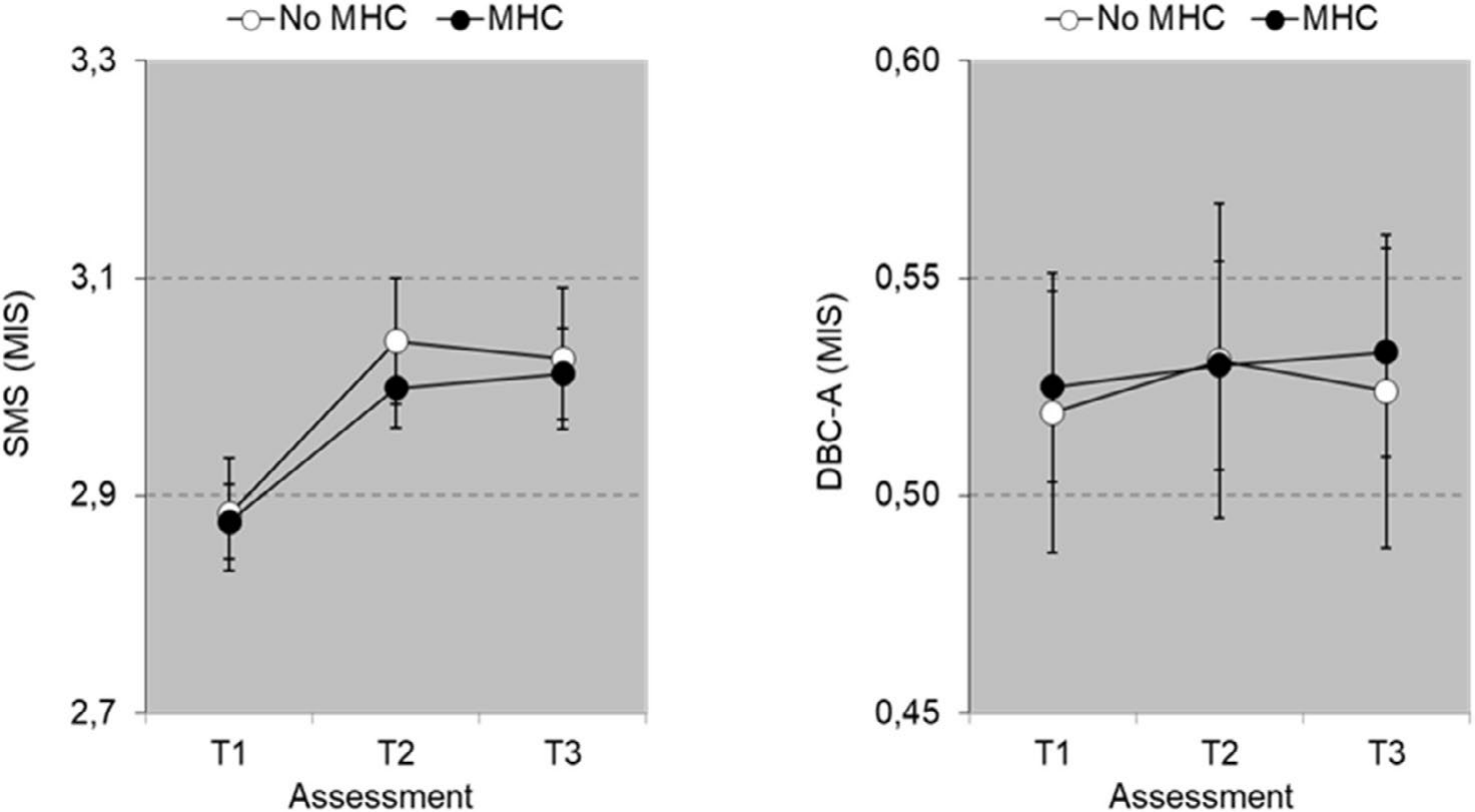
Quality of life (SMS total (MIS) ± SEM) and challenging behaviour (DBC‐A total (MIS) ± SEM) over time as a function of Group (No MHC vs. MHC).

The RM ANOVA on QoL (*N =* 82 MHC, *N =* 34 No MHC) resulted in a significant main effect of Time [*F*(2, 228) = 9.28, *p* < 0.001, *η*_p_^2^ = 0.075] and a significant first [*F*(1, 114) = 18.605, *p* < 0.001, *η*_p_^2^ = 0.140] and second contrast [*F*(1, 114) = 10.963, *p* = 0.001, *η*_p_^2^ = 0.088]. The effect of Group was not significant (*p* = 0.696), indicating a comparable level of QoL. The Group × Time interaction was not significant, overall and per contrast (0.573 < *p* < 0.925). These results indicate that QoL improved from T1 to T2 and this improvement was maintained at T3 and changes were independent of Group (see Figure 2, left panel).

The results of the hierarchical regression analyses, exploring the contribution of initial QoL, mental health and number of support goals to changes in QoL over time were as follows. For the group without MHC, Step 1, entering QoL at T1 into the model, was statistically significant [*F*(1, 39) = 9.935, *p* = 0.003], explaining 20.3% of the variance in QoL from T1 to T2. Step 2, adding cognitive functioning to the model, was significant [*F*(2, 38) = 10.347, *p* < 0.001], explained variance increased to 35.5%. Step 3, including total number of goals, was significant [*F*(3, 37) = 12.977, *p* < 0.001], explaining 51.3% of the variance. The increase (*F* change) at Step 2 and 3 were significant [*F*(1, 38) = 8.777, *p* = 0.005]. and [*F*(1, 37) = 12.159, *p* = 0.001] respectively. In the final model, the standardised coefficient *β* was −0.618 (*p* < 0.001) for QoL at T1, 0.433 (*p* = 0.002) for mental health and −0.407 (*p* = 0.001) for total number of support goals. This result indicates that lower QoL at T1, higher cognitive functioning and less support goals are associated with an increase in QoL.

For the group with MHC, Step 1, entering QoL at T1 into the model was statistically significant [*F*(1, 94) = 18.294, *p* < 0.001], explaining 16.3% of the variance. Step 2 adding cognitive functioning to the model [*F*(2, 93) = 10.897, *p* < 0.001], was significant, explained variance increased not significant to 19% (*p* = 0.083). Step 3, including total number of goals, was significant [*F*(3, 92) = 7.297, *p* < 0.001], explained variance increased not significant to 19% (*p* = 0.587). In the final model, the standardised coefficient *β* was −0.427 (*p* < 0.001) for QoL at T1, indicating that lower QoL at T1 was associated with an increase in QoL at T2, while cognitive functioning and total number of support goals did not significantly add to the model.

### 3.3 Changes in Challenging Behaviour Over Time

The RM ANOVA on challenging behaviour over time (*N =* 94 MHC, *N =* 43, No MHC) did not result in any significant main or interaction effect, overall and per contrast (0.630 < *p* < 0.908). Adding cognitive function as a covariate did not result in any significant effect. These results indicate that severity of challenging behaviour did not differ between groups with and without mental health classification and challenging behaviour did not show any significant change over time (see Figure 2, right panel).

### 3.4 Contrasting Groups That Showed Actually Improvement vs. Deterioration in Quality of Life Over Time

**FIGURE 3:**
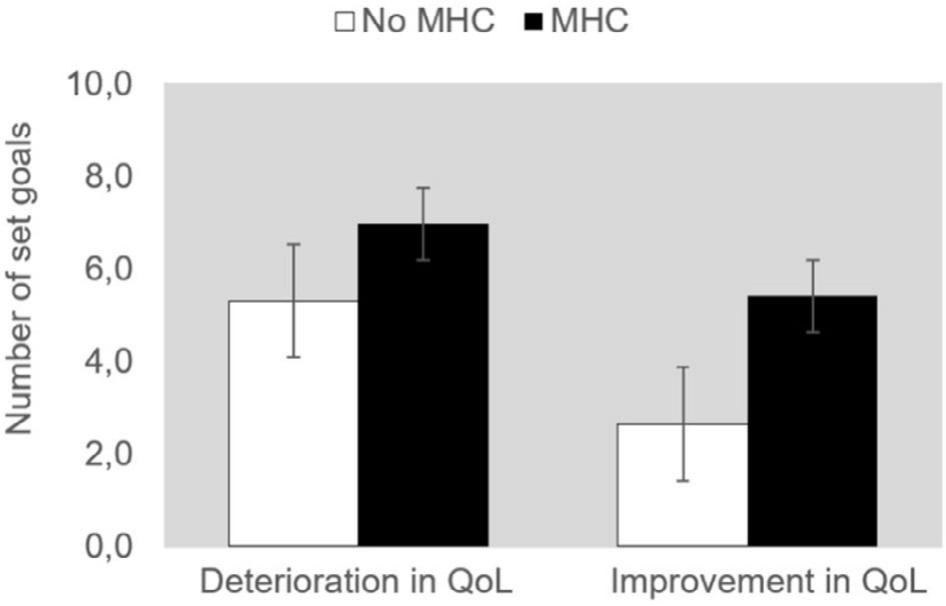
Number of goals as function of group and changes in quality of life over time.

The two‐way ANOVA exploring the number of set goals in the groups showing actual improvement versus deterioration in QoL over time, contrasting those with and without MHC, resulted in significant effects of Type of Change (positive vs. negative) [*F*(1, 133) = 5.094, *p* = 0.026, *η*_p_^2^ = 0.037] and Group (MHC vs. No MHC) [*F*(1,133) = 5.631, *p* = 0.019, *η*_p_^2^ = 0.041]. The interaction Type of Change × Group was not significant (*p* = 0.551). These results (see Figure 3) indicate that in the group improving in quality of life, less goals were set than in the group deteriorating in quality of life and that in the group with mental health classification more goals were set than in the group without mental health classification. These results confirm the notion that setting fewer goals is associated with higher quality of life, irrespective of whether mental health was classified or not.

A MANOVA comparing the number of goals set per domain of QoL between the groups that showed improvement vs. deterioration in QoL over time, irrespective of MHC, showed a significant difference on the domains of physical well‐being [F(1,137) = 6.534, *p* = 0.012, *η*_p_^2^ = 0.046] and interpersonal relations [*F*(1, 137) = 5.816, *p* = 0.017, *η*_p_^2^ = 0.041], indicating that in the group improving in quality of life, less goals were set in the domains of physical well‐being and interpersonal relations. On the other six domains, no significant differences in the number of set goals between groups were found (0.159 < *p* < 0.557).

A post hoc analysis to find out whether a lesser number of goals were set on these domains *because* the level of physical well‐being and interpersonal relations in that group was higher than in the group that deteriorated in QoL revealed that this was not the case for both physical well‐being (*p = 0*.449) and interpersonal relations (*p =* 0.081).

## 4 Discussion

This longitudinal study explored whether classification of mental health problems according to the DSM, contributes to quality of life in individuals with intellectual disabilities and severe challenging behaviour. The assumption was that the use of mental health classification supports the selection of effective support and intervention goals, reflected in improvement in quality of life and decrease of challenging behaviour. The results showed that the presence of a mental health classification did not improve quality of life or challenging behaviour outcomes over time. Regardless of whether a mental health classification was available, quality of life improved and intensity of challenging behaviour did not change over a three‐year period. Significantly more goals were listed in the individual support plans when a mental health classification was present, in particular regarding the enhancement of self‐determination, personal development, emotional well‐being and social inclusion.

The findings show that information from mental health classifications may be of limited use to select successful interventions in this particular intensive support context. One might argue that the limited contribution of DSM classification may be due to the difficulty to translate a mental health classification into practical goals that support quality of life or better targeted care in this specific population, especially since mechanisms of problem behaviour may be atypical due to complex developmental disorders (Baksh et al. 2023; Fletcher et al. 2009; Holden and Gitlesen 2004).

The finding that challenging behaviour did not improve over time corroborates the literature and once more illustrates the persistence of challenging behaviour as the outcome of a complex interaction of underlying individual and contextual characteristics (Baksh et al. 2023; Embregts et al. 2019; Perera et al. 2024; Smith et al. 2022; Thompson et al. 2022). Interventions must be tailored to suit individual needs and for effective implementation, communication and trust between all stakeholders (clients, caregivers, professionals) (Royston et al. 2023). To accomplish this consistently in daily practice is difficult. It is also possible that support and treatment provided are more focused on improving quality of life and less on decreasing challenging behaviour.

More goals were listed in individual support plans when a mental health classification was present. Interestingly, in individuals without a mental health classification, setting fewer goals was associated with improvement in quality of life over time. In individuals with a mental health classification this relation was non‐existent. This suggests that while a mental health classification may lead to a clearer picture of personal needs, it also appears to lead to setting more intervention targets, especially in terms of promoting self‐determination, personal development, emotional well‐being and social inclusion. This setting of more support goals, however, does not help to improve quality of life or to diminish challenging behaviour. In individuals without mental health classification, a lower number of goals is related to more positive change in quality of life. Since the level of intelligence is different for the comparison groups, general conclusions should be made cautiously, because different levels of intellectual functioning might be associated with different dynamics in the relation between support and quality of life. Focusing on those whose quality of life *actually* improved versus those whose quality of life deteriorated over time, setting fewer goals is associated with an improvement in quality of life regardless of a mental health classification. The results suggest that focusing the limited time and resources available on less support goals is associated with an increase in quality of life. Another possible explanation of this finding is that prioritising support goals is more important. The contribution of a mental health classification might even be counterproductive, since it seems to stimulate the increase of number of intervention goals.

Among the individuals without a mental health classification, improvement in quality of life over time was preceded by lower quality of life, higher cognitive functioning and fewer support goals at T1, explaining 51.3% of the variance. The effect of level of quality of life at T1 may be partly accounted for by ‘regression to the mean’, which is probably not depending on intervention. Importantly, higher cognitive functioning appears to relate to higher receptivity to treatment, which is compatible with previous studies suggesting greater difficulty in enhancing quality of life when personal skills are limited (Esteban et al. 2021; Gur 2018). For the group with mental health classification, changes in quality of life over time were only predicted by quality of life at T1, explaining just 16.3% of the variance in the change in quality of life over time, while cognitive functioning and number of intervention goals did not add to this result. These results may also suggest that lower cognitive skills might be associated with more difficulty to select support goals that align with the cognitive abilities. In other words, the possibilities for effective interventions might be more limited and less well developed and elaborated for lower cognitive level.

To our knowledge, the impact of evidence‐based knowledge about effective intervention provided by mental health classification on decision making in the design of contextual support and intervention for individuals with intellectual disability and severe challenging behaviour has not been previously studied. The longitudinal design plus the large number of participants can be considered a strength of our study. An unavoidable limitation is the use of proxy instruments, as for a vast majority of participants self‐report is not an option. Also, given missing data, unequal group sizes and the number of statistical tests performed, results should be treated with caution.

In summary, for individuals with intellectual disability that need 24/7 intensive support to navigate daily life demands because of severe challenging behaviour, the use of mental health classifications led to setting more support goals, but was not associated with better outcomes. Overall, independent of the presence of mental health classification, setting fewer goals was associated with improvement in quality of life, in particular for individuals with higher cognitive functioning. These findings support the hypothesis that, given the limited time and resources, greater effectiveness may be achieved with less support goals, that is, by prioritising goals. Additionally, results also suggest that individuals with higher cognitive functioning are more receptive to treatment.

## Funding

The SCORE project is funded by a consortium of specialised service organisations in care for individuals with intellectual disabilities and challenging behaviour in the Netherlands, that is, Ipse de Bruggen, De Hartekamp Groep, Cordaan and Ons Tweede Thuis.

## Ethics Statement

Ethical approval for the study was granted by the ethics committee of the Faculty of Social and Behavioural Sciences, Clinical Neurodevelopmental Sciences, Leiden University (ECPW‐2015/094) and by the ethical committee of the largest participating health care organisation (Ipse de Bruggen).

## Consent

Informed consent to participate in the SCORE project was obtained from either participants themselves, if they had the mental capacity to take an informed decision or (in most cases) from their legal representatives.

## Conflicts of Interest

The authors declare no conflicts of interest.

## Data Availability

After closure of the study, data are available from the authors upon reasonable request.
